# Divergent Human Papillomavirus Associated with Recurrent Respiratory Papillomatosis with Lung Involvement

**DOI:** 10.1128/genomeA.00474-13

**Published:** 2013-07-11

**Authors:** Hang Yuan, Dan Zhou, Jingang Wang, Richard Schlegel

**Affiliations:** Department of Pathology, Georgetown University Medical School, Washington, DC, USA

## Abstract

A divergent human papillomavirus (HPV), isolated from a lung lesion of a patient with recurrent respiratory papillomatosis, was fully cloned, sequenced, and genetically characterized. DNA analysis revealed that the HPV contained a 10.4-kb genome, with a duplication of 2,493 bp that includes partial L1-long control region (LCR)-E6-E7-partial E1 sequences.

## GENOME ANNOUNCEMENT

Recurrent respiratory papillomatosis (RRP) is the most common benign neoplasm of the larynx (1). Most cases are due to infection by human papillomavirus type 6 (HPV-6) or HPV-11 that is acquired at birth during passage through an HPV-infected birth canal. HPV-11 is associated with a more aggressive clinical course, including involvement of the lung (2). When it progresses into the lung parenchyma (less than 1% of cases), there are no effective therapies and it is almost invariably fatal (3). The precise cellular and/or viral alterations that promote lung invasion are unknown, although it has been speculated that HPV mutations may contribute to the progression (4).

A male patient with a 19-year history of recurrent respiratory papillomatosis developed progressive, bilateral tumor invasion of the lung parenchyma (5). DNA analysis revealed that the metastatic pulmonary tumor cells contained a 10.4-kb genome. Rolling circle amplification (RCA) was used to amplify episomal HPV DNA. The amplified viral genome was cloned into the vector pUC19 and sequenced from two directions using primer walking. The genome was then cloned into a pUC19 vector and primer walking enabled sequencing of the entire viral genome from both directions. Analysis of the viral sequence was performed using ABI 3730xl DNA-analyzing instruments for capillary electrophoresis and fluorescent dye terminator detection. Vector NTI Advance 10 software (Invitrogen) was used to assemble the sequence contigs containing high-quality trace files. Sequencing data revealed that the mutant HPV-11 genome contained 10,424 bp (GenBank accession number JN644141) due to duplication of 2,493 bp that includes partial L1-long control region (LCR)-E6-E7-partial E1 sequences.

Our data suggest a link between the duplication of the HPV-11 promoter and E6/E7 oncogenes and the clinical aggressiveness of the tumor in RRP. The present report describes the first sequence-confirmed duplication of the LCR-E6-E7 region in HPV. An earlier study had noted duplications of HPV-11 genomes in RRP that had progressed to squamous cell carcinoma and metastasized (6). Although there was no information on the viral DNA sequence, the published restriction enzyme digestion pattern was exactly the same as the HPV-11 DNA in the present patient. The similarity of the mutant viral genomes suggests that a similar mechanism or selection force is responsible for this genetic modification. It is well established that the E6 and E7 genes of the “high-risk” HPVs play major roles in cell immortalization, transformation, and carcinogenesis. Detection of intragenomic duplication in viral genomes in RRP might be predictive of a poor clinical outcome, and additional studies are warranted to determine if this mutation converts benign HPV genomes to more aggressive phenotypes.

### Nucleotide sequence accession number.

The complete genome sequence of HPV-11 associated with lung progression is available in GenBank under accession number JN644141.
